# Improving Children’s Menus in Community Restaurants: Best Food for Families, Infants, and Toddlers (Best Food FITS) Intervention, South Central Texas, 2010–2014

**DOI:** 10.5888/pcd11.140361

**Published:** 2014-12-24

**Authors:** Sylvia Hurd Crixell, BJ Friedman, Deborah Torrey Fisher, Lesli Biediger-Friedman

**Affiliations:** Author Affiliations: BJ Friedman, Deborah Torrey Fisher, Lesli Biediger-Friedman, School of Family and Consumer Sciences, Texas State University, San Marcos, Texas.

## Abstract

**Background:**

Approximately 32% of US children are overweight or obese. Restaurant and fast food meals contribute 18% of daily calories for children and adolescents aged 2 to 18 years. Changing children’s menus may improve their diets. This case study describes Best Food for Families, Infants, and Toddlers (Best Food FITS), a community-based intervention designed to address childhood obesity. The objective of this study was to improve San Marcos children’s access to healthy diets through partnerships with local restaurants, removing sugar-sweetened beverages, decreasing the number of energy-dense entrées, and increasing fruit and vegetable offerings on restaurant menus.

**Community Context:**

San Marcos, Texas, the fastest growing US city, has more restaurants and fewer grocery stores than other Texas cities. San Marcos’s population is diverse; 37.8% of residents and 70.3% of children are Hispanic. Overweight and obesity rates among school children exceed 50%; 40.3% of children live below the poverty level.

**Methods:**

This project received funding from the Texas Department of State Health Services Nutrition, Physical Activity, and Obesity Prevention Program to develop Best Food FITS. The case study consisted of developing a brand, engaging community stakeholders, reviewing existing children’s menus in local restaurants, administering owner–manager surveys, collaborating with restaurants to improve menus, and assessing the process and outcomes of the intervention.

**Outcome:**

Best Food FITS regularly participated in citywide health events and funded the construction of a teaching kitchen in a new community building where regular nutrition classes are held. Sixteen independent restaurants and 1 chain restaurant implemented new menus.

**Interpretation:**

Improving menus in restaurants can be a simple step toward changing children’s food habits. The approach taken in this case study can be adapted to other communities. Minimal funding would be needed to facilitate development of promotional items to support brand recognition.

## Background

Although the increase in incidence rates of overweight and obesity in children has been leveling, approximately 32% of US children and adolescents aged 2 to 19 are overweight or obese (1). Dietary intake is a modifiable determinant of body weight. Ecological models depict potential influences on diet that range from personal factors (eg, biological, lifestyle) to broader realms of influence (eg, social, environmental, public policy) (2). Of the broader influences, food consumed away from home, which falls in the environmental realm, has become prominent. Children and adolescents aged 2 to 18 obtain 34% of daily calories from food consumed outside the home, 18% of which comes from fast food and full-service restaurants (3). Compared with home-cooked meals, foods consumed away from home are higher in calories, fat, sodium, and sugar and contain fewer essential nutrients (4). Meals away from home often include sugar-sweetened beverages and lack fruits and vegetables (4). Improving offerings on children’s menus in restaurants may improve dietary intake and thereby combat obesity (5).

Some community interventions have attempted to improve the nutrition profile of restaurant menus. For example, Shape Up Somerville worked with local restaurants to reduce portion sizes, add fruits and vegetables, and offer reduced-fat dairy products (6). Steps to a Healthier Salinas targeted taquerias to encourage the development and promotion of healthier foods (7). In San Antonio, Texas, ¡Por Vida! involved registered dietitians working with local restaurants to identify menu items that met Dietary Guidelines for Americans (8,9).

The objective of this study was to improve San Marcos children’s access to healthy foods by working with local restaurants to help them improve their menus by removing sugar-sweetened beverages, decreasing the number of energy-dense entrées, and increasing fruit and vegetable offerings. This article describes Best Food FITS, a community program dedicated to combatting obesity by engaging restaurants to improve children’s menus in a community with high rates of childhood obesity.

## Community Context

The Best Food FITS intervention was funded by a $150,000 grant from the Texas Department of State Health Services Nutrition, Physical Activity, and Obesity Prevention Program. This study was conducted in San Marcos, a city in south central Texas located about halfway between the major metropolitan areas of Austin and San Antonio. For the second consecutive year, San Marcos has been designated the fastest-growing city in the country (10). The population is ethnically diverse. Approximately 37.8% of the 54,000 residents (11) and 70.3% of school children enrolled in the San Marcos Consolidated Independent School District (CISD) are Hispanic (12). The poverty level in San Marcos is over 130% higher than the national average; 40.3% of children in San Marcos live below the poverty level (11,12). San Marcos school children have higher rates of overweight and obesity than children across the state (36.6%) and nation (31.3%) (13). According to FITNESSGRAM data, 52% of 5th, 7th, and 9th graders in the San Marcos CISD are overweight or obese (14). Dietary practices of children in this area that may be associated with excess body weight include high intake of sugar-sweetened beverages and low intake of fruits and vegetables (15,16).

San Marcos is a major exit on Interstate Highway 35 and has considerably more restaurants per capita than the rest of the state and fewer grocery stores per capita (11). There is no city restaurant association. 

## Methods

We chose 3 obesity-prevention focus areas identified by the Texas Nutrition, Physical Activity, and Obesity Prevention Program for the Best Foods FITS intervention: increasing intake of fruits and vegetables, decreasing intake of sugar-sweetened beverages, and decreasing intake of energy-dense foods (16). Best Foods FITS began in spring 2010 and consisted of 6 elements: 1) developing a Best Food FITS brand with a recognizable logo and graphics; 2) introducing Best Food FITS as a new partner in the established health community; 3) collecting and reviewing existing children’s menus in San Marcos restaurants; 4) asking owners or managers about restaurant practices and capacity; 5) collaborating with participating restaurant owners or managers to improve children’s menus; and 6) assessing the process and outcomes of the intervention. The Texas State University institutional review board approved all aspects of this project.

### Best Food FITS branding

**Figure 1:**
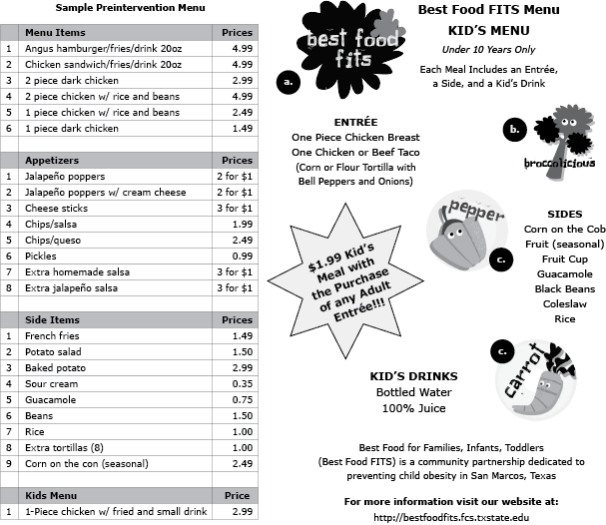
Sample Preintervention and Best Food for Families, Infants, and Toddlers (Best Food FITS) Menu from a Mexican Food Restaurant in San Marcos, Texas. The Best Food FITS menu includes the a) Best Food FITS logo, b) the popular Broccolicious character, and c) other characters. All graphics were created by the marketing department at Texas State University.

To foster brand recognition, we collaborated with the university marketing department to develop a Best Food FITS logo and cartoon graphics of vegetables designed to appeal to children. The logo and graphics were used on Best Food FITS promotional items (eg, T-shirts, bumper stickers, health fair posters) and menus (Figure 1). A Best Food FITS logo decal was later affixed to doors of participating restaurants. The brand was advertised throughout the community through articles in local newspapers, a television interview, social media, and community health partner organizations.

### Launching Best Food FITS

We introduced Best Food FITS by hosting 2 meetings to which we invited leaders from all community organizations representing the diverse San Marcos population, including the local Special Supplemental Nutrition Program for Women, Infants, and Children agency, Head Start, the county health department, the city housing authority, the local food bank, the Hays County Extension office, Central Texas Medical Center, the local gardening group, the farmers market, and the public housing authority. The owner of a landmark local restaurant was also invited. The primary agenda of these meetings, attended by 10 to 12 community leaders, was to brainstorm on how to effectively advance the Best Food FITS mission of improving children’s diets to combat obesity. The Best Food FITS research team also met individually with stakeholders.

### Assessing existing menus

**Figure 2:**
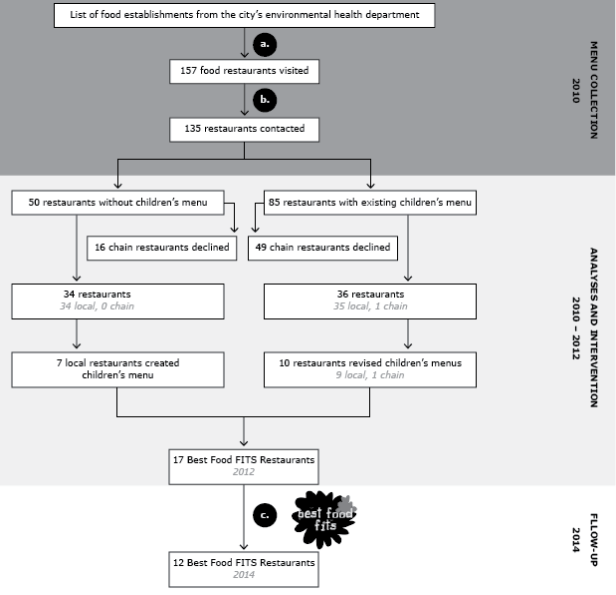
Timeline for the Best Food for Families, Infants and Toddlers (Best Food FITS) Restaurant Intervention, San Marcos, Texas, 2010–2014. Excluded establishments were a) food stores, bars, bowling alleys and b) coffee shops and restaurants going out of business. In 2014, c) 2 Best Food FITS restaurants had gone out of business and 3 had withdrawn.

In summer 2010, the city’s environmental health department provided a list of local establishments that served food. After eliminating food stores and restaurants that did not serve children, we visited the remaining 157 restaurants (or accessed fast food menus online) to collect available children’s menus. We then eliminated coffee shops and restaurants that were going out of business, yielding a final list of 135 restaurants, 85 of which had children’s menus (Figure 2). We asked the restaurant staff to clarify beverage and side options that were not clearly described, and 3 registered dietitians then classified menu items as follows: beverages as sugar-sweetened (eg, sodas, flavored milk, specialty drinks), nonnutritive (eg, diet sodas, unsweetened tea), or healthy (eg, milk, 100% juice); entrées as unhealthy (fried, cheesy, greasy, or fatty) or healthy, depending on content and preparation; and sides as unhealthy (fried, fatty, or starchy) or healthy vegetables, or as fruit. Desserts were classified as healthy if they were fruit; otherwise, they were classified as unhealthy (Table 1). Children’s menus were also scored using the Children’s Menu Assessment system, which scores 21 items, such as availability of healthy entrées, free refills on sugar-sweetened beverages, healthy fruit and vegetable offerings, and the practice of brand marketing on menus (17).

**Table 1:** Menu Item Categorization, Analyses of Children’s Menus in San Marcos, Texas, Restaurants, 2011

Menu Category^a^	Items^b^
**Entrées**	
Healthy entrées	Meats (grilled, baked, or lean)
	Salads (nonfatty entrée salads)
	Vegetable platters
Fried, cheesy, greasy, or fatty entrées	Any fried entrées
	Macaroni and cheese
	Alfredo or cream sauce
	Creamy or cheesy soups
	Chicken nuggets, tenders, or fried strips
	Corn dogs
	Pizzas
	Grilled cheese sandwiches
	Cheesy Mexican food (burritos, chimichangas, enchiladas, quesadillas, chalupas)
**Fruits**	
Healthy fruits	Apple slices
	Applesauce
	Bananas
	Fruit (cups, salads)
	Mandarin oranges
	Pineapple slices
Sweetened or fried fruits	Cinnamon apples
	Fried apples
	Fried/tempura bananas
**Vegetables**	
Healthy vegetables	Raw vegetables
	Salads (side, green, house salads)
	Steamed vegetables
Fried or fatty vegetables	Fatty salads (coleslaw, Caesar)
	Fried okra
	Fries (French, steak, sweet)
	Onion rings
	Tater tots
Starchy vegetables	Corn (kernels, corn on the cob)
	Potatoes (mashed, roasted, baked)
**Other sides**	
Legumes	Beans (pinto, black)
	Chickpeas/hummus
	Black-eyed peas
Miscellaneous sides	Breads
	Sauces
	Rice
	Chili
	Dumplings
	Yogurt
	Cheese
Fried, cheesy, greasy, or fatty sides	Macaroni and cheese
	French fries with cheese
	Chips
	Fried rice
**Desserts**	
Healthy	Unsweetened fruits
Sweetened or fried desserts	Bananas Foster
	Cakes
	Cheesecake
	Cookies
	Cupcakes
	Fried fruit
	Fruit cobbler
	Ice cream (plain and fried)
	Kolaches
	Milkshakes, malts
**Beverages**	
Sugar-sweetened beverages: sodas	All soft-drinks sweetened with sugar
Sugar-sweetened beverages: nonsoda	Flavored milks
	Fruit punch, fruit drink
	Hot chocolate
	Lemonades, limeades
	Slushes
	Smoothies (with added sweetener)
	Sports drinks
	Sweetened/flavored teas (raspberry, blackberry)
Nutritive	Juice (100%)
	Milk (cow, nondairy)
	Smoothies (with no added sweetener)

a Categorization methods similar to those used in Krukowski et al (17).

b All menu items appeared on children’s menus in San Marcos, Texas, restaurants.

### Changing children’s menus

We initially planned to inform this intervention by first engaging restaurant owners and managers through focus groups. Owners declined, citing time constraints, so we changed our approach. Working in teams of 2, researchers attempted to schedule a 20- to 30-minute meeting with owners or managers of the 135 restaurants by calling (up to 15 times) or visiting if calls were unsuccessful. When managers of chain restaurants declined, citing corporate policy, we attempted to contact corporate offices by e-mail, telephone, and via a letter of endorsement from the Texas Department of State Health Services, asking for participation.

When meetings with owners or managers were successfully scheduled, researchers 1) described the project, 2) obtained informed consent, 3) administered a survey asking about the use of children’s menus and the owner or manager’s thoughts about children’s menus, 4) explained the health impact of child obesity by using a flyer, and 5) asked for the restaurant’s participation to improve existing children’s menus or to develop new menus meeting Best Food FITS guidelines.

We designated 3 levels for restaurant menus: gold, silver, and bronze. The gold menus were required to have at least 3 fruit or vegetable options, and most entrées had to be classified as healthy. The silver menus were required to have at least 2 fruit or vegetable options, and most entrées had to be healthy. Bronze menus were required to have at least 1 fruit or vegetable, and many of the entrées had to be healthy. All levels required omission of sugar-sweetened beverages from menus. Incentives to participate included free advertising through the Best Food FITS website (http://bestfoodfits.fcs.txstate.edu/) and via community stakeholders, social media, newspaper, and radio interviews.

The research teams worked iteratively with owners or managers either to revise existing or develop new children’s menus. This process began with a meeting to develop a hand-written draft menu based on the owner’s or manager’s preferences, incorporating items already present on existing menus. After the meeting, researchers created a colorful menu using Microsoft PowerPoint, shared the menu with the owner and manager in person or via e-mail, and modified as needed. We provided laminated or paper copies of the new, approved menus as requested by the owner or manager. Once menus were in place, participating restaurants were asked to display a Best Food FITS decal at the restaurant entrance. We provided restaurants with table tents, give-away T-shirts, coloring placemats, bibs, cups, and reusable grocery bags with the Best Food FITS logo and graphics for promoting the program to their patrons. We frequently contacted participating restaurants to provide support and to adjust the new children’s menus upon request.

### Evaluating outcomes

We evaluated the launching of Best Food FITS by documenting our participation in community health activities. Assessment of the restaurant intervention included a count of the restaurants that agreed to offer Best Food FITS menus. Also, the content (eg, counts of sugar-sweetened beverages, fruits and vegetables, healthy entrées), cost, and children’s menu assessment scores of preintervention (n = 85) and Best Food FITS (n = 17) menus were compared by using independent-sample *t* tests with SPSS, Version 22 (IBM Corp). In 2014, we conducted a postintervention assessment to determine if Best Food FITS menus were still in use. Owners or managers in 4 participating restaurants administered a survey to customers with children who used Best Food FITS menus. The 10-question patron survey, adapted from a nonchain restaurant intervention survey (18), asked whether customers noticed the decal on entering the restaurant, noticed the Best Food FITS options when ordering, and to what extent their ordering decisions were influenced by healthy menu options.

## Outcome

**Launching Best Food FITS**. Best Food FITS has been invited to at least 4 community-wide health and wellness events (eg, hospital, university, and city health fairs) per year since 2011, at which we have hosted a booth advertising our purpose, engaged participants in an interactive demonstration involving guessing the sugar content of popular sugar-sweetened beverages, and distributed promotional items such as bumper stickers, T-shirts, and reusable grocery bags. Adults and children alike enjoyed T-shirts depicting Broccolicious (character shown in Figure 1). Pictures of people wearing the T-shirt can be found on our Facebook page (www.facebook.com/groups/121423094574163/), which now has over 300 members. The Best Food FITS logo and graphics have contributed to our community presence.

Discussions with stakeholders led to recognition of shared interest in the need for community cooking classes. When we learned that the local public housing authority was in the final stages of planning construction of an adult learning center at Chapultepec Homes, we were able to leverage grant funds to install 4 working kitchens in that facility. Chapultepec is part of The Family Self-Sufficiency Program (http://www.smpha.org/node/76), a national housing program that promotes employment and improving the resources of low-income families. The Best Food FITS logo is on the permanent signage for the Chapultepec Adult Learning Center. Since 2011, we and other community stakeholders, such as the local food bank and afterschool programs, have used the center to engage over 300 local residents each year in community nutrition and hands-on cooking classes that emphasize fruits and vegetables. We have ensured the sustainability of this collaboration by incorporating student-led delivery of classes at Chapultepec Homes into the university nutrition and foods undergraduate curriculum.

Further evidence of the intervention’s community reach includes funding by a local philanthropic group in 2012 to expand Best Food FITS to promote healthy food environments in local childcare centers and to work with parents of children in childcare to improve home food environments. The Best Food FITS childcare and parent initiatives were conducted in 2013 and 2014. Additionally, Best Food FITS was invited to become a member of the San Marcos Healthy City Task Force, a new city organization to promote community health.

**Best Food FITS restaurant initiative**. Initial assessment of children’s menus in 85 San Marcos restaurants from 2010 through 2011 indicated typical American staples such as fast food, pizza, sandwiches, barbeque, and steak; in addition, 17% offered Mexican food exclusively. Notably, children’s menus offered an average of 8 varieties of sugar-sweetened beverages and 3 entrées classified as fried, cheesy, greasy, or fatty (Table 2). French fries were an automatic side on 44% of menus. The average number of healthy entrées and vegetable sides were 2.02 and 1.08, respectively.

**Table 2:** Comparison of Children’s Menus in San Marcos, Texas, Before and After the Best Food FITS Intervention, 2010–2014

Characteristic	Restaurants Before Intervention (N = 85)	Best Food FITS Restaurants (N = 17)	
**Restaurant type^a^**	**N (%)^b^**	**N (%)^b^**	
Fast food	28 (32.9)	0	
Full service/fast casual	28 (32.9)	17 (100)	
Sandwich/deli	8 (14.0)	0	
Pizza	2 (3.5)	0	
Mexican or Tex-Mex	15 (26.3)	9 (52.9)	
American	22 (38.6)	4 (23.5)	
Asian	4 (7.0)	2 (11.8)	
BBQ or burger (nonchain)	3 (5.3)	1 (5.9)	
Italian	3 (5.3)	0	
Mediterranean	0	1 (5.9)	
**Menu options**	**Mean N (SD)**	**Mean N (SD)**	***P* value^c^**
**No. sugar-sweetened beverages options**			
Sodas	4.49 (2.06)	0	<.001
Other sugar-sweetened beverages (flavored milks, specialty drinks)	3.68 (3.70)	0	<.001
Total sugar-sweetened beverages	8.17 (4.04)	0	<.001
**No. entrées**			
Fried, cheesy, greasy, or fatty entrées	3.08 (2.27)	0.24 (0.66)	.04
Healthy entrées	2.02 (1.71)	4.12 (1.90)	<.001
**No. side dishes**			
Fried, cheesy, or greasy sides^d^	2.04 (1.60)	1.18 (0.95)	<.001
Fruit sides	0.42 (0.66)	1.35 (1.06)	.003
Fried, fatty, starchy vegetable sides	1.21 (1.13)	0.06 (0.24)	<.001
Healthy vegetable sides	1.08 (1.90)	3.24 (1.64)	<.001
**Meal cost (average cost of children’s meal), $**	4.07 (0.90)	4.53 (1.83)	.41
**Children’s Menu Assessment score^e^, total score**	1.85 (2.76)	8.53 (2.12)	< .001

Abbreviations: Best Food FITS, Best Food for Families, Infants and Toddlers; SD, standard deviation.

a Restaurant classification based on Children’s Menu Assessment (17).

b Percentages may not sum to 100% because of rounding.

c Independent-samples *t* test was conducted to compare averages for preintervention and Best Food FITS restaurants; based on the Bonferroni correction, differences between means were considered to be significant if *P* value was <.005 (19). All categories were significantly different except for fried, cheesy, greasy, or fatty entrées.

d Sides made of fat with grains or starchy vegetables (eg, macaroni and cheese, French fries).

e Children’s Menu Assessment total scores can range from −5 to 21 (17), with higher values indicating more healthy menus.

Of the 135 restaurants that we approached, 65 chain restaurants declined to participate. Of the 70 remaining restaurants not citing corporate restrictions, 7 restaurants agreed to create new children’s menus, and 10 (including one chain) volunteered to revise existing menus, yielding a total of 24% of local restaurants implementing Best Food FITS children’s menus in 2012 (Figure 2). A sample menu changed through the Best Food FITS intervention is included in Figure 1. During follow-up assessments in 2014, 12 restaurants were still using the menus. Reasons for attrition from the program included restaurant closures and disinterest.

Compared with preintervention menus, the Best Food FITS menus offered significantly more healthy items and significantly fewer unhealthy items in most food and beverage categories that were assessed (Table 2). Also, the average Children’s Menu Assessment score for Best Food FITS menus was significantly higher than for preintervention menus (Table 2). Although we were initially surprised that the average cost of a kid’s meal on the new menus was not significantly higher than that of pre-intervention menus, an assessment of the cost of entrées in full-service chain restaurants in Little Rock, Arkansas, also indicated that more healthy entrées on children’s menus were not more expensive than less healthy entrées (20). Analysis of 35 patron surveys indicated that more than 25% of respondents were aware of the Best Food FITS decal in the window or entryway upon arriving at the restaurant, almost 50% noticed the Best Food FITS options on the children’s menu, and 51% reported that nutrition was a very important factor when deciding their food choices.

Gathering input from owners and managers provided some insight about management’s perspectives about changing menus (Table 3). For example, of the 61 respondents, about one-third agreed that sugar-sweetened beverages were the most profitable item on the menu, and 15% said they did not stock fruits and vegetables, suggesting significant barriers to improving menus. Conversely, most respondents agreed that sugar-sweetened beverages were bad for health and also agreed that restaurants should serve healthy foods and provide alternatives to sugar-sweetened beverages.

**Table 3:** Perspectives of 61 Restaurant Owners and Managers About Children’s Menus Preintervention, Best Food FITS, San Marcos, Texas, 2010

Survey Question	Yes, N (%)^a^	No, N (%)^a^	Neutral or Not Applicable, N (%)^a^
**Restaurant practices and capacity**			
Are there barriers to removing sugar-sweetened beverages from your children’s menu?	10 (16.4)	27 (44.3)	24 (39.3)
Are sugar-sweetened beverages the most profitable item on your menu?	19 (31.1)	39 (63.9)	3 (4.9)
Does your restaurant currently stock fruits and vegetables?	52 (85.2)	9 (14.8)	0
Do you feel there would be problems adding any (or more) fruits and vegetables to your menu?	15 (24.6)	41 (67.2)	5 (8.2)
Is there not enough kitchen space (to store more fruits and vegetables)?	8 (13.1)	50 (81.9)	3 (4.9)
Is there not enough refrigeration/freezer space (to store more fruits and vegetables)?	15 (25.6)	41 (67.2)	5 (8.2)
Is your establishment a chain restaurant?	24 (39.3)	37 (60.7)	0
**Opinions about food and health**			
Are sugar-sweetened beverages “bad” for health?	50 (81.9)	10 (16.4)	1 (1.6)
Should restaurants provide alternatives to sugar-sweetened beverages?	59 (96.7)	2 (3.3)	0
Should restaurants provide healthy foods?	52 (85.2)	9 (14.8)	0
Is it important for restaurants to serve healthy foods?	54 (88.5)	7 (11.5)	0
Is it important to your customers to have healthy foods?	46 (75.5)	14 (23.0)	1 (1.6)
Have your customers previously demanded healthy foods?	35 (57.4)	25 (41.0)	1 (1.6)
Have you previously tried to make healthy changes in your restaurant?	33 (54.1)	26 (42.6)	2 (3.3)
Do you feel confident in choosing healthy foods?	52 (85.2)	0	9 (14.8)

Abbreviations: Best Food FITS, Best Food for Families, Infants and Toddlers.

a Percentages may not sum to 100% because of rounding.

This community intervention provides insight into how to engage restaurant owners and managers to voluntarily change their menus. The most important lesson learned was to be patient and persistent, because many establishments never answered their phones or returned messages. The work-around strategy was to have teams visit those restaurants, frequently during the afternoon, to try to establish contact. In a few cases, a surprise encounter converted an elusive owner into an enthusiastic participant. We learned it was important to take time to build the relationship. Enthusiastic undergraduate and graduate university student research teams frequented restaurant establishments, often eating meals with owners and devoting time to establishing rapport. During the menu development process, Best Food FITS teams and owners or managers collaborated iteratively to create and individualize menus. Owners and managers were more open to change if new menus included items already available on the restaurants’ menus. This strategy probably contributed to the cost neutrality of the menu changes. Importantly, we learned not to assume we could predict which restaurants would participate. For example, while we expected and were gratified to engage some of the landmark local restaurants that are frequented by the university community, we were surprised with the successful recruitment of many smaller Mexican food restaurants. In fact, approximately half of the original participating Best Food FITS restaurants were from these neighborhood establishments. The Broccolicious T-shirts and other branded incentives were valued by restaurant owners and managers and may have contributed to recruitment success. Finally, as an engaged stakeholder, the owner of a prominent local restaurant set a positive tone by being the first to adopt a Best Food FITS menu.

This intervention had limitations. With the exception of 1 chain restaurant, all corporately owned establishments declined to participate, limiting the number of Best Food FITS menus in the community. Through direct communication or through the owner and manager survey, we learned that for some restaurants, sugar-sweetened beverages were important drivers of revenue and that their removal would pose a financial burden. Best Food FITS developed a work-around by requiring that sugar-sweetened beverages not appear on children’s menus at participating restaurants but could still be offered on adult menus. This compromise appealed to many owners and managers. This strategy was also adopted by the ¡Por Vida! initiative in San Antonio (9).

## Interpretation

Best Food FITS is now a recognized brand in San Marcos. Despite a few challenges, including the absence of a city restaurant association to serve as a point of contact, 24% of nonchain restaurant owners and managers proved to be valuable partners in improving their children’s menus. Their participation demonstrated concern for their community and for the children who live in it. The approach described in this case study can easily be adapted to other communities. Although funding is needed to generate promotional items, the actual menu development can be done at minimal cost through nutrition programs in universities or in collaboration with local hospital dietitians. Improving the usual menu options, such as including fruit and vegetable sides, can be an important step in changing restaurant norms and in building a less obesogenic community for children (5).

## References

[1] Ogden CL , Carroll MD , Kit BK , Flegal KM . Prevalence of childhood and adult obesity in the United States, 2011-2012. JAMA 2014;311(8):806–14. 10.1001/jama.2014.732 24570244 PMC4770258

[2] Story M , Kaphingst KM , Robinson-O'Brien R , Glanz K . Creating healthy food and eating environments: policy and environmental approaches. Annu Rev Public Health 2008;29:253–72. 10.1146/annurev.publhealth.29.020907.090926 18031223

[3] Poti JM , Popkin BM . Trends in energy intake among US children by eating location and food source, 1977–2006. J Am Diet Assoc 2011;111(8):1156–64. 10.1016/j.jada.2011.05.007 21802561 PMC3148484

[4] Batada A , Bruening M , Marchlewicz EH , Story M , Wootan MG . Poor nutrition on the menu: children’s meals at America’s top chain restaurants. Child Obes 2012;8(3):251–4 [Formerly Obesity and Weight Management]. 22799552 10.1089/chi.2012.0016

[5] Anzman-Frasca S , Dawes F , Sliwa S , Dolan PR , Nelson ME , Washburn K , Healthier side dishes at restaurants: an analysis of children’s perspectives, menu content, and energy impacts. Int J Behav Nutr Phys Act 2014;11:81–92. 24996545 10.1186/1479-5868-11-81 PMC4102063

[6] Economos CD , Folta SC , Goldberg J , Hudson D , Collins J , Baker Z , A community-based restaurant initiative to increase availability of healthy menu options in Somerville, Massachusetts: Shape up Somerville. Prev Chronic Dis 2009;6(3): http://www.cdc.gov/pcd/issues/2009/jul/08_0165.htm. Accessed August 1, 2014. 19527574 PMC2722387

[7] Hanni KD , Garcia E , Ellemberg C , Winkleby M . Targeting the taqueria: implementing healthy food options at Mexican American restaurants. Health Promot Pract 2009;10(2, Suppl):91S–9S. 10.1177/1524839908331268 19454755

[8] Sosa ET , Biediger-Friedman L , Banda M . Associations between a voluntary restaurant menu designation initiative and patron purchasing behavior. Health Promot Pract 2014;15(2):281–7. 10.1177/1524839912469535 23271720

[9] Biediger-Friedman L , Sosa E , Shields K , Shutt A . A voluntary approach to improve menu options in restaurants through a local collaborative partnership. Texas Public Health Journal 2014;66(1):11–4 [Por Vida study]. http://connection.ebscohost.com/c/articles/94732871/voluntary-approach-improve-menu-options-restaurants-through-local-collaborative-partnership. Accessed December 1, 2014.

[10] South, West have fastest-growing cities, Census Bureau reports; three of top 10 are in Texas capital area. Washington (DC): US Census Bureau; 2014. http://www.census.gov/newsroom/press-releases/2014/cb14-89.html#. Accessed July 1, 2014.

[11] State and County QuickFacts. San Marcos, Texas. Washington (DC): US Census Bureau. http://quickfacts.census.gov/qfd/states/48/4865600.html. Accessed July 31, 2014.

[12] Public Schools Explorer. San Marcos CISD. Austin (TX): The Texas Tribune; 2014. http://www.texastribune.org/public-ed/explore/san-marcos-cisd/. Accessed July 31, 2014.

[13] Data Resource Center for Child and Adolescent Health. US State Rankings Map. Overweight/obese children. Portland (OR): The Child and Adolescent Health Measurement Initiative; 2014. http://childhealthdata.org/browse/rankings/maps?s=84. Accessed August 1, 2014.

[14] Mapping FITNESSGRAM. http://www.reshapingtexas.org/fitnessgram. Accessed July 31, 2014.

[15] Self W , Roberts R . Consolidated independent school district, high school youth risk behavior surveillance survey report. Youth Risk Behavior Surveillance Survey. Kyle (TX): Hays Consolidated Independent School District; 2012.

[16] Nutrition, Physical Activity and Obesity Prevention Program. Austin (TX): Texas Department of State Health Services; 2014. https://www.dshs.state.tx.us/obesity/NPAOPprogrampage.shtm. Accessed July 31, 2014.

[17] Krukowski RA , Eddings K , Smith West D . The children's menu assessment: development, evaluation, and relevance of a tool for evaluating children’s menus. J Am Diet Assoc 2011;111(6):884–8. 10.1016/j.jada.2011.03.018 21616202

[18] Nothwehr FK , Snetselaar L , Dawson J , Schultz U . Promoting healthy choices in non-chain restaurants: effects of a simple cue to customers. Health Promot Pract 2013;14(1):132–8. 10.1177/1524839912437368 23048009 PMC3956304

[19] Maxwell SE , Delaney H . Designing experiments and analyzing data: a model comparison perspective. 2nd ed. Mahwah (NJ): Lawrence Erlbaum; 2004.

[20] Krukowski RA , West D . No financial disincentive for choosing more healthful entrées on children’s menus in full-service restaurants. Prev Chronic Dis 2013;10E94. 10.5888/pcd10.120266 23742942 PMC3682812

